# Chronic appendiceal perforation leading to cecal–abdominal wall fistula long misdiagnosed as recurrent groin abscess: a rare case report

**DOI:** 10.3389/fmed.2026.1901981

**Published:** 2026-10-22

**Authors:** Jun Li, Wen-Na Liu, Zheng-Hong Jiang, Hai-Bo Xiong, Li-Bin Deng, Li-Hao Deng

**Affiliations:** 1 Department of Gastrointestinal Hernia and Abdominal Wall Surgery, Affiliated Hospital of Chengdu University, Chengdu, Sichuan, China; 2 Department of Laboratory Medicine, Sichuan Provincial People's Hospital, School of Medicine, University of Electronic Science and Technology of China, Chengdu, China

**Keywords:** cecal–abdominal wall fistula, chronic appendiceal perforation, psoas abscess, rare case, recurrent groin abscess

## Abstract

**Background:**

Chronic appendiceal perforation is extremely rare in adults, and secondary cecal–abdominal wall fistula is even more scarcely reported in the literature. We present a 64-year-old man with a 20 + year history after retroperitoneal abscess drainage and 10 + years of recurrent right groin abscess, ultimately diagnosed as chronic appendiceal perforation with cecal–abdominal wall fistula.

**Case presentation:**

The patient underwent open retroperitoneal abscess drainage more than 20 years ago. Subsequently, he developed recurrent right groin pain, erythema, swelling, and fever, repeatedly diagnosed as right iliac fossa abscess requiring multiple incisional drainages, but the condition persisted. Contrast-enhanced abdominal CT and sinography (iohexol) showed a thickened appendix with ill-defined borders from the right psoas muscle, psoas abscess, a tubular structure extending to the right inguinal subcutaneous tissue, and hyperdense contents in the cecum and appendiceal lumen. Preoperative diagnoses: appendiceal perforation and cecal–abdominal wall fistula. Laparoscopic right hemicolectomy, intra-abdominal abscess drainage, abdominal wall sinus tract excision, and adhesiolysis were performed. Intraoperatively, the appendiceal base, body, and adjacent cecum were retroperitoneal, forming a 3.5 × 3.0 cm abscess with a palpable tract to the right inguinal defect. Pathology showed chronic and acute inflammation of the intestinal mucosa with serosal fibrous proliferation, consistent with a cecal fistula. Postoperative paralytic ileus resolved with conservative management. Long-term follow-up revealed no recurrence.

**Conclusion:**

Chronic appendiceal perforation is a rare cause of cecal–abdominal wall fistula, which can present as recurrent groin abscess for years. Clinicians should be aware of this entity. Contrast-enhanced CT combined with sinography aids preoperative diagnosis, and complete surgical resection is curative.

## Introduction

Acute appendicitis is a common surgical emergency, but chronic appendicitis and chronic appendiceal perforation are clinically rare. Chronic appendiceal perforation often lacks typical signs of acute peritonitis; the inflammation can slowly spread retroperitoneally, involve the psoas muscle, form an abscess, and even penetrate the abdominal wall to create an enterocutaneous fistula. When such lesions present as recurrent groin abscess, they are easily misdiagnosed as primary groin infection, postoperative sinus tract, or iliac fossa abscess. A search of PubMed databases reveals extremely few case reports of chronic appendiceal perforation leading to cecal–abdominal wall fistula, especially with a disease course exceeding 20 years. We report such a case to highlight its clinical features, diagnostic keys, and surgical principles.

## Case presentation

A 64-year-old man was admitted with a chief complaint of “recurrent right inguinal abscess for more than 10 years” and a history of retroperitoneal abscess drainage performed more than 20 years earlier at another hospital. After the initial open retroperitoneal drainage, he experienced recurrent right groin pain, erythema, swelling, and fever. He was repeatedly diagnosed with “right iliac fossa abscess” and underwent multiple incision and drainage procedures. Despite these interventions, he continued to have spontaneous skin breakdown and purulent discharge from the right inguinal region. The clinical course of the patient is summarized in Figure 1.

**Figure 1:**
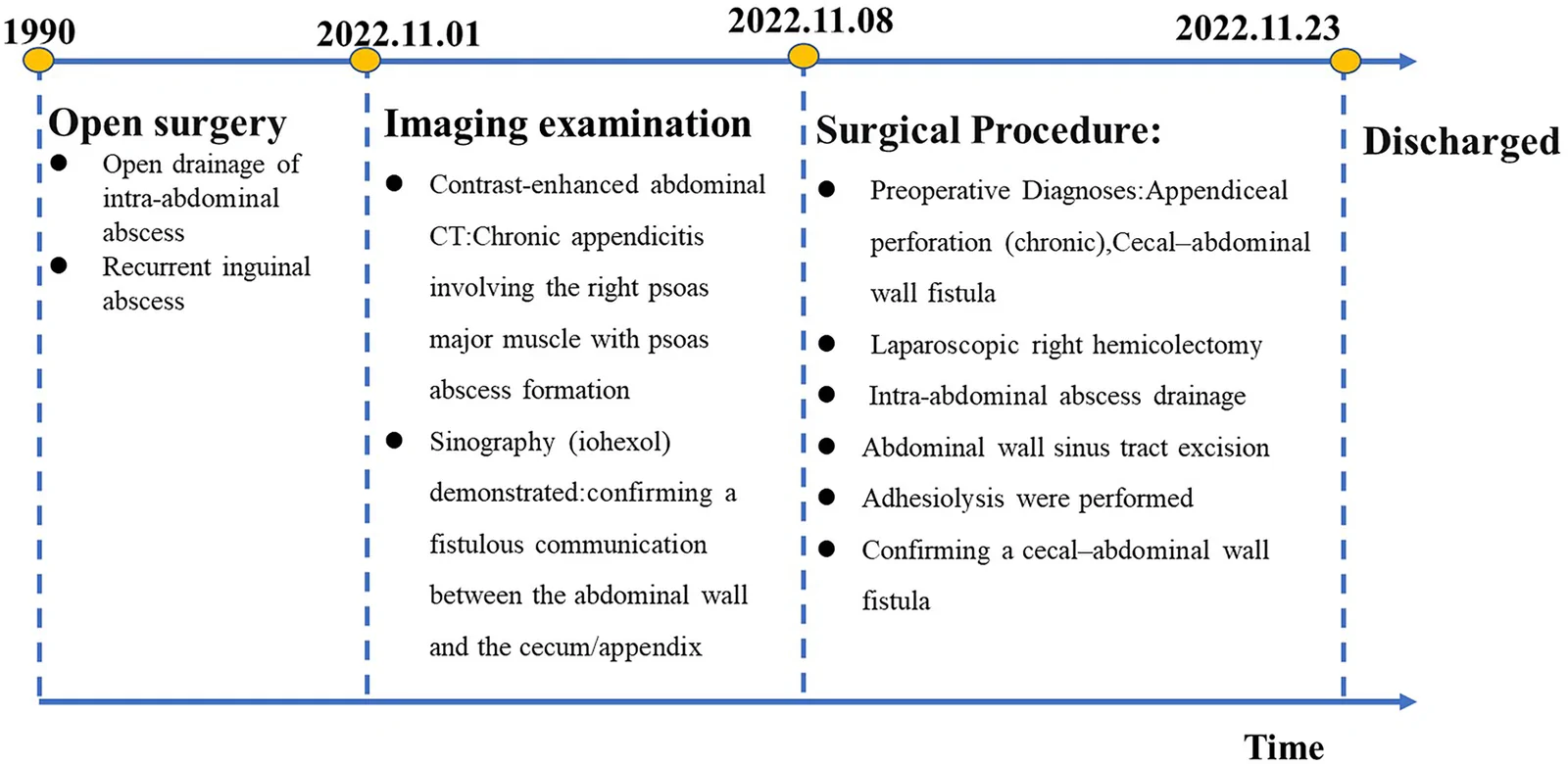
Clinical timeline of the patient's presentation, diagnosis, surgical management, and recovery.

The detailed records and imaging data of the initial retroperitoneal abscess drainage and the subsequent multiple inguinal abscess excisions, which were performed at multiple other hospitals more than 20 years ago, are unavailable; therefore, the early pathophysiological process cannot be fully reconstructed.

Physical examination revealed old surgical scars and a cutaneous opening in the right inguinal region with purulent discharge on compression. Laboratory tests showed mild leukocytosis and elevated C-reactive protein. Contrast-enhanced abdominal CT revealed a thickened appendix with an indistinct border from the right psoas muscle, periappendiceal fat stranding and a small amount of fluid, swelling of the right psoas muscle with patchy hypodense areas and nodular enhancement after contrast, and a tubular structure extending from the lower aspect to the right inguinal subcutaneous tissue with adjacent skin irregularity, leading to an imaging diagnosis of chronic appendicitis involving the right psoas muscle with associated peritonitis, psoas abscess formation, and possible right lower quadrant sinus tract (Figure 2). Sinography after iohexol injection through the right inguinal cutaneous defect demonstrated a thickened appendix with an indistinct border from the psoas muscle, periappendiceal fat stranding, psoas swelling with hypodense areas, a tubular structure extending to the groin, and importantly, hyperdense contents within the cecum and appendiceal lumen (indicating contrast entering the cecal and appendiceal lumen), thereby confirming an abnormal communication from the ileocecal region to the abdominal wall (Figure 3). On November 4, 2022, gastroscopy revealed chronic atrophic gastritis (C2, active). On November 4, 2022, colonoscopy revealed a tortuous colon, with no colonic ulcers and no colonic neoplasms. Colonoscopy showed no cecal perforation or colonic diverticula. Preoperative gastroscopy and colonoscopy ruled out ulcerative colitis, Crohn's disease, and colorectal malignancy. Preoperative microbiological culture of the purulent discharge from the right inguinal abscess grew Escherichia coli, supporting an enteric source.

**Figure 2:**
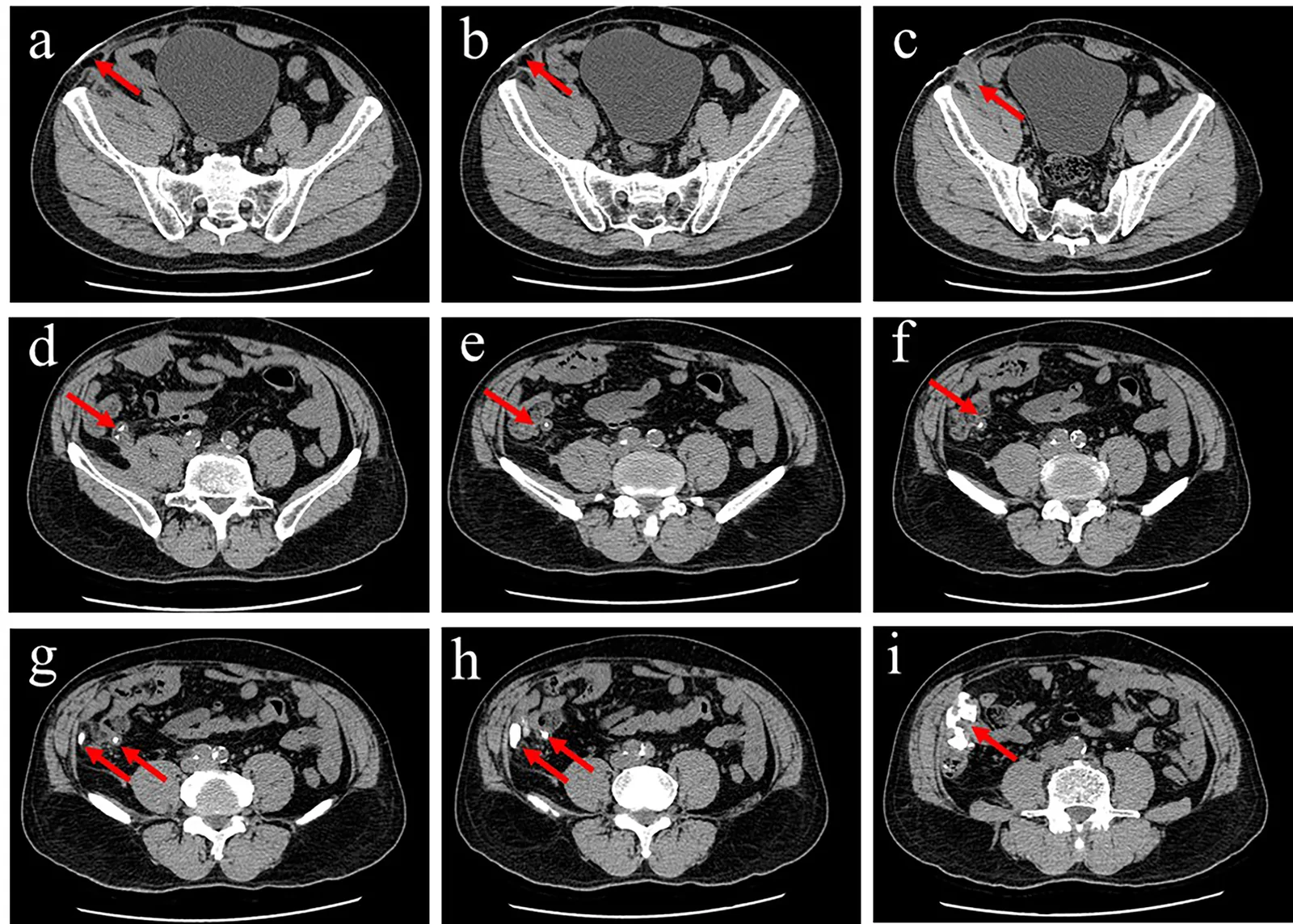
Retrograde sinography through the external opening of the right inguinal sinus tract. (**a–i**) Sequential images after iohexol injection via the cutaneous orifice of the right inguinal region. The contrast medium rapidly opacified the sinus tract, then retrogradely entered the appendix (arrows), and finally flowed into the ascending colon (arrowheads), confirming a fistulous communication between the abdominal wall and the cecum/appendix. This finding was consistent with chronic appendiceal perforation complicated by a cecal–abdominal wall fistula.

**Figure 3:**
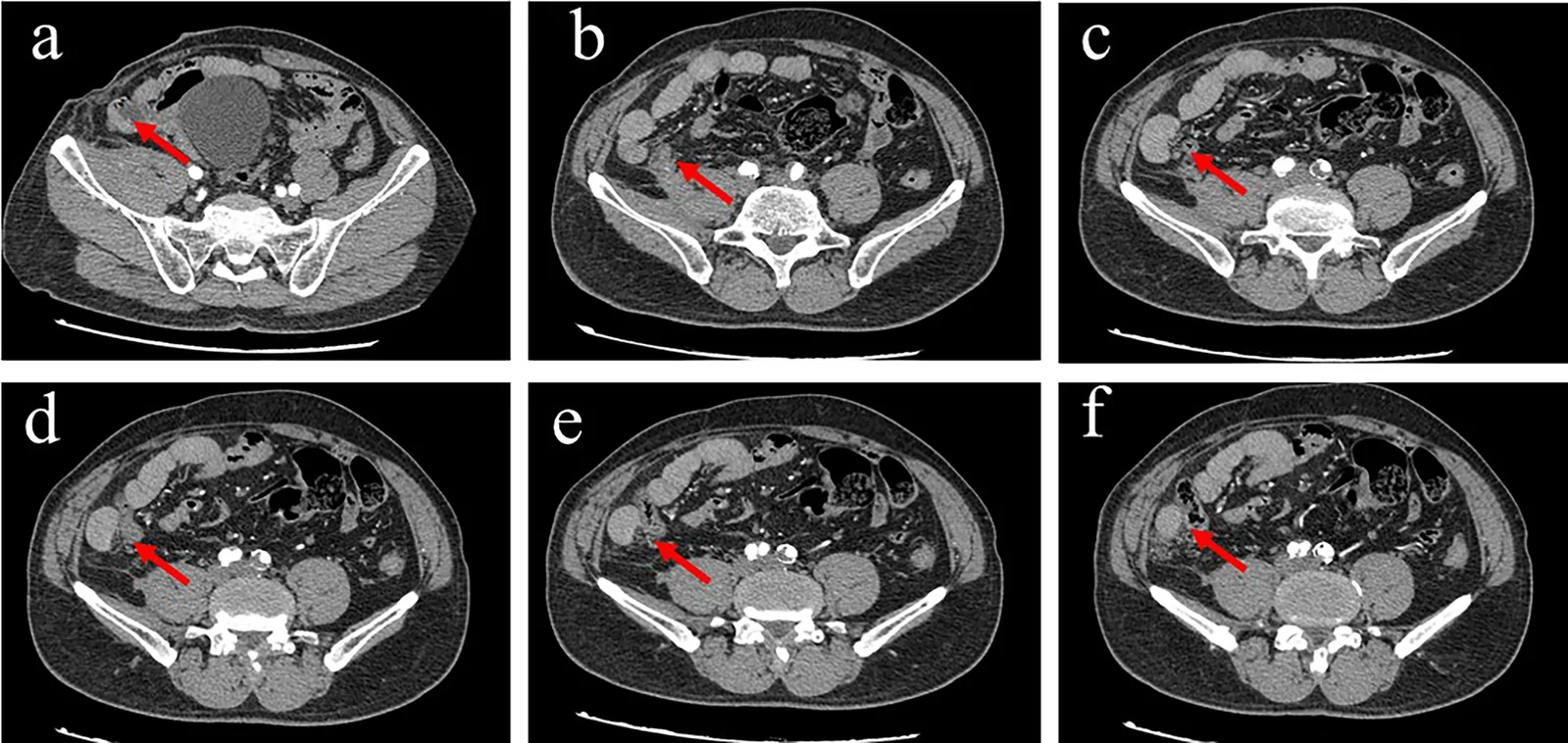
Contrast-enhanced abdominal CT findings. (**a–f**) The images demonstrate chronic appendicitis with involvement of the right psoas major muscle. There is periappendiceal fat stranding and localized fluid collection, indicating surrounding peritonitis. The right psoas muscle is swollen with hypodense areas, consistent with psoas abscess formation. In addition, a sinus tract is visible in the right lower quadrant, extending toward the inguinal region. These findings are compatible with chronic appendiceal perforation complicated by periappendiceal peritonitis, psoas abscess, and abdominal wall sinus tract.

The preoperative diagnoses were chronic appendiceal perforation and cecal–abdominal wall fistula. Under general anesthesia, the patient underwent laparoscopic right hemicolectomy, intra-abdominal abscess drainage, abdominal wall sinus tract excision, and adhesiolysis. Intraoperatively, the omentum and part of the small bowel were adherent below the previous incision, and the small bowel in the right lower quadrant was matted together. The base of the appendix was located retroperitoneally; the appendiceal body and a portion were also retroperitoneal, Intraoperative exploration revealed a perforation measuring 5 mm in diameter at the base of the appendix. The adjacent cecum was likewise fixed retroperitoneally and, together with the posterior abdominal wall, formed an abscess measuring approximately 3.5 × 3.0 cm. A tract was palpable from the distal part of the abscess leading to the right inguinal cutaneous defect, confirming a cecal–abdominal wall fistula. The right colon (including the ileocecal region and appendix), the abscess wall, and the abdominal wall sinus tract were completely excised, and adhesions were lysed.

Postoperatively, the patient was managed with fasting, antibiotics, intravenous fluids, analgesia, acid suppression, and nutritional support. He developed paralytic ileus (abdominal distension, no flatus or defecation) on postoperative day 3, which significantly improved after nasogastric decompression, continued antibiotics, and fluid resuscitation. Histopathological examination of the resected right colon, appendix, abscess wall, and fistula tract showed chronic inflammation with acute exacerbation, focal mucosal atrophy and shedding, congestion and edema of the lamina propria, and fibrous tissue proliferation on the serosal surface. There were no atypical cells, no mucin lakes, no invasive glandular structures, and no morphological evidence of mucinous neoplasm or cecal adenocarcinoma. There were no granulomas, no crypt architectural distortion, no basal plasmacytosis, and no transmural lymphoid aggregates suggestive of Crohn's disease. Therefore, neoplastic and inflammatory bowel disease-related etiologies were formally excluded. The final pathological diagnosis was a cecal fistula secondary to chronic appendiceal perforation. No malignancy was identified (Figure 4).

**Figure 4:**
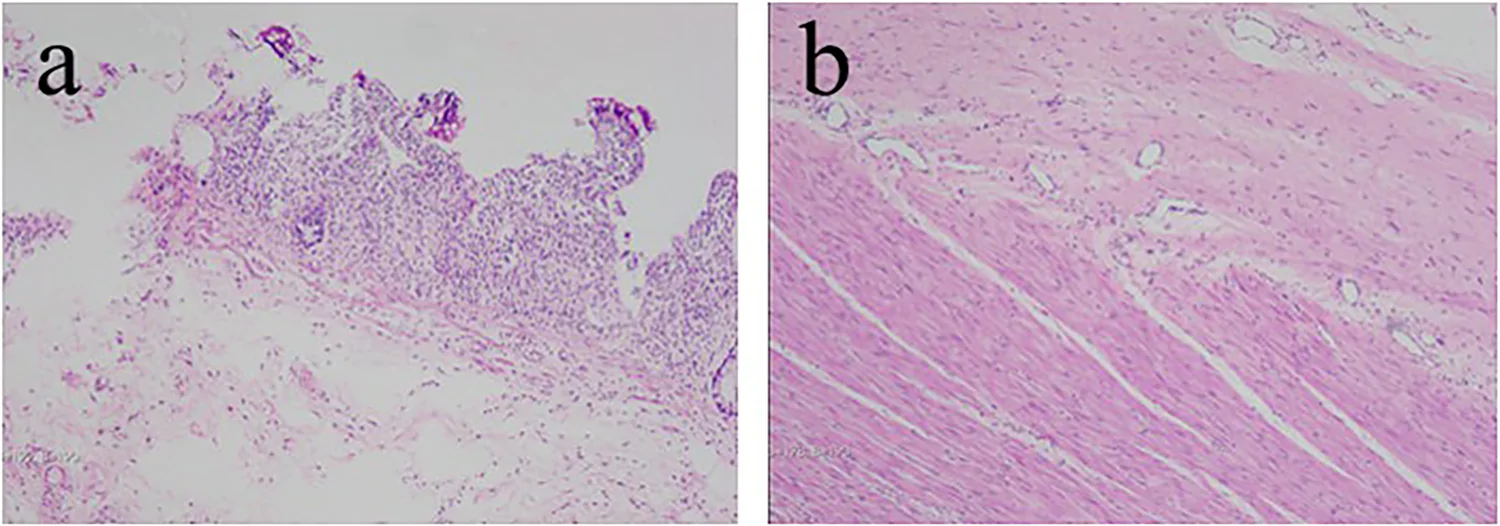
Postoperative histopathological findings. The images (**a, b**) Microscopic examination of the resected right colon and fistulous tract shows chronic inflammation of the intestinal mucosa with acute exacerbation, characterized by focal mucosal atrophy and shedding, congestion and edema of the lamina propria, and fibrous tissue proliferation on the serosal surface. These findings are consistent with a cecal fistula. No evidence of malignancy is identified.

### Follow-up and outcomes

Long-term follow-up (>2 years) revealed no recurrence of abdominal wall abscess or groin infection, and the patient's quality of life was excellent.

### Patient perspective

The patient reported that recurrent right groin swelling and purulent discharge over more than 10 years had substantially affected his daily life, causing repeated hospital visits and drainage procedures. He expressed anxiety about recurrence before surgery. After laparoscopic right hemicolectomy and fistula excision, he experienced no recurrence during follow-up and reported excellent quality of life. He provided written informed consent for publication of this case report and accompanying images.

## Discussion

Chronic appendiceal perforation in adults is extremely uncommon (1), and its rarity can be attributed to several pathophysiological and clinical factors. First, acute appendiceal perforation typically leads either to rapid containment by the omentum and adjacent structures (forming a localized abscess) or to diffuse peritonitis and sepsis (2–7). These acute or subacute presentations dominate clinical practice, whereas a truly chronic, indolent perforation that results in a persistent low-grade fistula or occult drainage tract is exceptionally rare (8). Second, the appendix has a narrow lumen and a terminal arterial supply; once transmural necrosis occurs, the resulting defect is usually large, precipitating an acute catastrophic event rather than a slow leak (9–12). Third, chronic perforation often requires specific anatomical conditions, such as a retroperitoneal appendix. In such settings, inflammatory contents spread into the retroperitoneal space (e.g psoas, iliac fossa, perirenal space) without causing signs of peritonitis, thereby allowing a protracted, misdiagnosed course that may eventually manifest as a psoas abscess or abdominal wall fistula (13–15). Fourth, no widely accepted diagnostic criteria exist for chronic appendiceal perforation; most reported cases are diagnosed only after complications (abscess, fistula) develop, and large series indicate that chronic perforation accounts for less than 1% of all appendiceal perforations. Finally, a literature search reveals only sporadic case reports of chronic appendiceal perforation presenting with retroperitoneal abscess or enterocutaneous fistula (16–18), and those with a disease course exceeding one or two decades are even more exceptional (19). The present patient had a disease course exceeding 20 years with recurrent groin abscess, ultimately diagnosed as chronic appendiceal perforation with cecal–abdominal wall fistula—a finding that is exceptionally rare in the literature (19). A PubMed search using the keywords “chronic appendiceal perforation” and “cecal-abdominal wall fistula” (up to 2026) revealed only sporadic case reports, most with disease duration of less than one year. Thus, the extremely long course and insidious presentation of this case are highly instructive for clinicians.

Due to congenital or acquired retroperitoneal location of the appendix and cecum, chronic inflammation spread posteriorly to the psoas muscle, forming a psoas abscess (13, 20–24). The abscess then tracked along the psoas fascia inferiorly and eventually penetrated the groin subcutaneous tissue, creating a spontaneous abdominal wall fistula (25, 26). Because the inflammation was confined to the retroperitoneum, the patient never developed peritonitis but only recurrent groin drainage. This mechanism explains why repeated local drainage failed—the underlying cause was a chronic perforation in the cecum.

Imaging is central to diagnosis (27). Contrast-enhanced CT clearly shows the appendix morphology, psoas involvement, abscess, and the fistulous tract (28–30). Sinography directly identifies the internal opening by injecting contrast through the cutaneous defect; in this case, contrast entering the cecum and appendiceal lumen provided direct evidence of a cecal–abdominal wall fistula. The hyperdense contents in the cecum and appendix likely represent fecaliths or concentrated contrast, further supporting chronic perforation.

Because the etiology is a chronically perforated cecum and appendix, simple drainage or sinus tract excision inevitably leads to recurrence (31). Complete en-block resection of the involved bowel segment (right hemicolectomy), abscess cavity, and entire fistulous tract is the only curative approach. Laparoscopic surgery, even with dense adhesions, is feasible and offers advantages of less trauma and faster recovery. The postoperative paralytic ileus, a common complication after severe retroperitoneal infection (32–34), resolved with conservative management and did not require reoperation (35).

Previous reports of appendicitis-related psoas abscess are mostly secondary to acute appendicitis or appendiceal phlegmon (36–39). Cecal–abdominal wall fistula due to chronic perforation is far rarer. The unique features of this case include a disease course exceeding 20 years, a clinical presentation limited to recurrent groin abscess without typical appendicitis symptoms, preoperative sinography that directly confirmed the internal opening in the cecum, and successful laparoscopic radical resection. These features provide important diagnostic considerations for clinicians.

In patients presenting with a chronic inflammatory mass involving the ileocecal region and a long-standing fistula, mandatory differential diagnoses include appendiceal or cecal mucinous neoplasms, cecal adenocarcinoma, and Crohn's disease. In our patient, preoperative contrast-enhanced CT and sinography showed no discrete mass, mucinous features, or typical neoplastic architecture. Intraoperatively, there was no peritoneal dissemination or tumor-like mass. Postoperative histopathology of multiple sections showed only chronic inflammation and fibrosis, without atypical cells, mucin lakes, invasive glandular structures, granulomas, crypt architectural distortion, or transmural inflammation. These findings formally excluded mucinous neoplasm, cecal adenocarcinoma, and Crohn's disease. The definitive diagnosis was therefore a cecal fistula secondary to chronic appendiceal perforation.

The protracted 20-year diagnostic delay in this case merits further reflection. Several healthcare-system and cognitive factors may have contributed. First, the initial retroperitoneal abscess drainage was performed at another hospital, and the patient subsequently sought care at multiple institutions for recurrent groin symptoms. This resulted in fragmented medical records and limited continuity of care, which may have prevented clinicians from appreciating the full longitudinal course of the disease. Second, because the presentation closely mimicked a common primary groin abscess or postoperative sinus tract, clinicians may have anchored on this initial diagnosis and repeatedly performed incision and drainage rather than pursuing cross-sectional imaging. Third, the intermittent nature of the symptoms and the temporary relief after each drainage procedure may have reinforced diagnostic momentum and reduced the perceived need for further evaluation. Fourth, limited access to contrast-enhanced CT or sinography in some earlier clinical settings may also have contributed to the delay. This case therefore illustrates the importance of avoiding anchoring bias, maintaining a broad differential diagnosis, and obtaining early cross-sectional imaging in patients with recurrent groin or iliac fossa abscesses, especially those with a history of abdominal or retroperitoneal infection. It also highlights the value of integrated care, complete medical record review, and multidisciplinary collaboration in preventing diagnostic delay and related patient safety events.

This study has several limitations. First, as a single-case report, the findings are descriptive and cannot be generalized to all patients with enterocutaneous fistulas. Second, detailed records and imaging from the initial retroperitoneal abscess drainage performed at another hospital more than 20 years ago were unavailable, which limits full reconstruction of the early pathophysiology. Third, although the patient's perspective is now included in accordance with CARE guidelines, no standardized patient-reported outcome measures were collected. Despite these limitations, this case highlights a rare and diagnostically challenging entity.

## Conclusion

Chronic appendiceal perforation can lead to cecal–abdominal wall fistula, which may present solely as recurrent groin abscess without signs of peritonitis for years. This rare entity is easily misdiagnosed. For patients with long-standing, recurrent groin abscess, especially those with a history of prior abdominal surgery or retroperitoneal infection, a colonic or appendiceal retroperitoneal source should be suspected. Contrast-enhanced CT combined with sinography is a reliable preoperative diagnostic tool. Laparoscopic complete resection of the involved bowel segment, abscess, and fistula achieves cure with excellent long-term outcomes.

## Data Availability

The original contributions presented in the study are included in the article/supplementary material, further inquiries can be directed to the corresponding author.
